# PTSD Biomarker Database: deep dive metadatabase for PTSD biomarkers, visualizations and analysis tools

**DOI:** 10.1093/database/baz081

**Published:** 2019-06-29

**Authors:** Daniel Domingo-Fernández, Allison Provost, Alpha Tom Kodamullil, Josep Marín-Llaó, Heather Lasseter, Kristophe Diaz, Nikolaos P Daskalakis, Lee Lancashire, Martin Hofmann-Apitius, Magali Haas

**Affiliations:** 1 Department of Bioinformatics, Fraunhofer Institute for Algorithms and Scientific Computing, Sankt Augustin 53754, Germany; 2 Cohen Veterans Bioscience, 1 Broadway, Cambridge, MA 02142, United States

## Abstract

The PTSD Biomarker Database (PTSDDB) is a database that provides a landscape view of physiological markers being studied as putative biomarkers in the current post-traumatic stress disorder (PTSD) literature to enable researchers to explore and compare findings quickly. The PTSDDB currently contains over 900 biomarkers and their relevant information from 109 original articles published from 1997 to 2017. Further, the curated content stored in this database is complemented by a web application consisting of multiple interactive visualizations that enable the investigation of biomarker knowledge in PTSD (e.g. clinical study metadata, biomarker findings, experimental methods, etc.) by compiling results from biomarker studies to visualize the level of evidence for single biomarkers and across functional categories. This resource is the first attempt, to the best of our knowledge, to capture and organize biomarker and metadata in the area of PTSD for storage in a comprehensive database that may, in turn, facilitate future analysis and research in the field.

## Introduction

Post-traumatic stress disorder (PTSD) is a common psychiatric disorder that occurs in some individuals after a traumatic event (13) and is diagnosed by mental health professionals based on the presentation of four symptom clusters—intrusions, avoidance, negative cognitions/mood and hyperarousal (1). PTSD pathophysiology is complex and affects multiple interconnected biological systems that regulate mental and physical health functions and are associated with PTSD’s clinical heterogeneity and diverse comorbidity profiles (2,5,9,11,14,15).

An extensive amount of research in PTSD has explored the utility of physiological markers as being discrete biomarkers of this disorder; however, no such putative biomarkers of PTSD have been identified to date based on the regulatory approval process for qualifying and validating biomarkers around specific clinical contexts of use (hereafter the term ‘biomarker’ will be used to refer to ‘physiological markers of disease’). In the PTSD literature, the types of physiological markers most commonly studied include neuroimaging and psychophysiological measures, behavioral and neurocognitive readouts and analytes measured in peripheral biofluids, such as blood and saliva at baseline or after psychological challenge (4,6,8,12,20). Fluid-based peripheral biomarkers may include inflammation indicators, hypothalamic pituitary adrenal axis mediators, neurosteroids and neurotransmitters, which have functional roles both in the peripheral and central nervous system, potentially enabling biologically meaningful inference with clinical utility (4).

The increasing amount of biomarker studies in all disease areas is paralleled by the growing number of meta-analyses that combine data from multiple studies to systematically derive common conclusions. However, the lack of disease-specific biomarker registries impedes the harmonization and integration of results from these studies, which often remain in the form of non-structured text, figures, tables or supplementary files. Organizing and storing this knowledge is essential to provide a comprehensive view of the biomarker landscape and to foster the discovery and development of diagnostics and treatments.

Along these lines, several biomarker databases have been recently developed that focus on specific disease domains, such as colorectal cancer (19), Alzheimer’s disease (10), tuberculosis (18) and liver cancer (3) to name a few. Furthermore, there are multiple resources that store and catalog biomarker information from multiple indications, such as the Online Mendelian Inheritance in Man (OMIM) (7), the cancer biomarker database (16) and the infectious disease database (17). These resources illustrate how biomarker information can be curated and harmonized and currently serve as hubs for biomarker research in their respective areas. Although biomarkers of PTSD—once identified—have the potential to improve patient outcomes, groups have yet to embark on similar efforts for a PTSD-specific database.

To address this, we are developing the comprehensive PTSD Biomarkers Database (PTSDDB), focusing on fluid-based biomarkers as a resource for bringing together published findings within the context of study design and related results. Organizing and contextualizing published information and data are critical to understand the level of confirmatory and contradictory evidence for single biomarkers. Overall, this work represents one first step to crossing the translational divide between basic science discovery and clinical implementation. Considering findings for single biomarkers in tandem with details around study design may offer insights into the robustness and replicability of studies. Here, we present the first version of the PTSDDB biomarker database that provides a comprehensive and interactive view of results from an extensive, systematic curation effort in over 100 PTSD-focused articles. We aim to continually build on this resource in the future to enable a better interpretation of the state of the field in biomarker research that supports formal meta-analyses around single biomarkers in PTSD.

**Table 1:** Types of information extracted from each manuscript and stored as entries in the PTSDDB. For each data category, extensive information was curated and stored as separate entries in the PTSDDB. For example, the Data Category “Biomarker” includes information on Biomarker name, HUGO ID or another acronym, gene symbol/identifier, and biomarker application (e.g., biomarkers for disease risk, patient stratification, diagnostic marker, predictive markers of disease severity or treatment response, and safety/toxicity biomarkers).

**Model/data category**	**Fields**
Publication	PubMed identifier, authors information (e.g. names, year of publication and geographical information)
Biomarker	Biomarker name, HUGO ID or other acronym, gene symbol, biomarker application. Protein, gene or miRNA biomarkers are coded using the HGNC nomenclature if possible. Small molecules nomenclatures are prioritized in the following order: ChEBI, PubChem and InChIKeys.
Time point	Study time point (e.g. 6 weeks post-trauma, 2 years post-trauma)
Approach panel details	Name, statistical method, cutoff used (e.g. *P*-value, False Discovery Rate, etc.), whether the biomarker was part of a panel, other analytes included, risk SNP, allele risk and additional notes on risk SNP
Numerical summary	Statistics (i.e. mean and standard deviation [SD]) about the biomarker measurements for primary indication, trauma-exposed controls, other central nervous system and healthy controls
Clinical instrument	Clinical instrument for primary indication, trauma in adult, childhood and lifetime (e.g. Clinician-Administered PTSD SCALE [CAPS], PTSD Checklist [PCL], Structured Clinical Interview for DSM-5 [SCID], etc.)
Clinical study	Type of study (e.g. cross-sectional, longitudinal), timeline, challenge type, treatment response study, number of subjects per indication (e.g. trauma-exposed PTSD, trauma-exposed controls, healthy controls, other indications)
Indication	Name and specifics of the condition (e.g. PTSD, childhood trauma, maternal PTSD)
Comorbidity	Name, comorbidity measurements (e.g. mean and SD)
Numerical readouts	Statistical details of each different group included in the study (e.g. mean, SD of the biomarker for primary indication or control)
Inclusion/exclusion criteria	The description of inclusion and exclusion criteria provided by each publication
Cohort name and demographic details	Details about the cohort (e.g. percent female plus the overall mean and SD in trauma-exposed PTSD vs trauma-exposed and healthy controls, mean age and SD age of subjects)
Ancestry	Ancestry details for Caucasian, African American and other ancestry of the cohort (e.g. percentage of each group included in the study)
Study findings	Direction of change of the biomarker in cases vs controls as specified by the study authors (e.g. increased, no change, decreased), specific circuit changes, notes and descriptions
Assay	Assay details: assay brand, probe, fluid, biological substrate, assay brand, assay limit detection and measurement units
Assay calculations	Mean and SD concentration (in primary indication, trauma-exposed controls, healthy controls and CNS controls), sigma combined and effect size
Statistical info	Mean, SD and variance, methylation change, *t* and *P*-value

## Materials and Methods

### Curation procedure and database content

#### Corpus selection

Articles included in the PTSDDB were compiled via two routes: recommendations and referrals from experts in the field and mining cited references from PTSD review publications. In general, publications included in this deep-dive database were original articles published from 1997 to 2017 that evaluated fluid-based biomarkers in humans, with a focus on PTSD patients vs control populations (e.g. healthy controls, trauma-exposed controls and/or patients with psychiatric disorders or other comorbidities). Exclusion criterion included publications that did not include a PTSD population, those that included PTSD patients but in the absence of fluid-based biomarkers, or that were preclinical studies.

#### Data extraction and quality assessment

The biomarker metadata information contained in the resulting 109 publications was manually extracted independently by five independent trained curators and added to a data model template. The spectrum of curated metadata covers many fields, including study design, demographics, study findings, assay information and statistical methods. Ultimately, three rounds of quality control (QC) were conducted to ensure the fidelity of the metadata. In each round of QC, the metadata was reviewed by a distinct curator; if inconsistencies were found, curators worked together to reach a consensus. While there exist other QC procedures ensuring the quality of the curated data such as inter-curator agreement, these involve significant time constraints as they require two distinct curators working in parallel to extract the same information. On the other hand, our QC procedure allowed us to include a larger amount of biomarker metadata while maintaining the quality of data curation. The data model template for metadata extraction was predefined based on the initial set of 10 articles. However, new metadata fields were added as necessary to accommodate new types of information being extracted from additional manuscripts.

### Database design and web application implementation

#### Database model

One challenge in data integration when curating disparate sources of information is organizing this knowledge according to a common schema, which greatly influences subsequent steps of data management and analysis. In order to structure the information comprised in the 193 columns of the curation template/worksheet, we designed a data model storing this information into 17 different models (e.g. publication, biomarker, clinical study, cohort metadata, etc.), which are represented as tables in the database (Table 1). Next, we implemented a parser of the curation template that populates the MySQL database and controls the quality of the worksheet by identifying duplicates, checking the syntax and normalizing terms. Finally, a web application integrated the database enabling users to query, visualize and analyze the curated content as illustrated in this manuscript.

**Figure 1:**
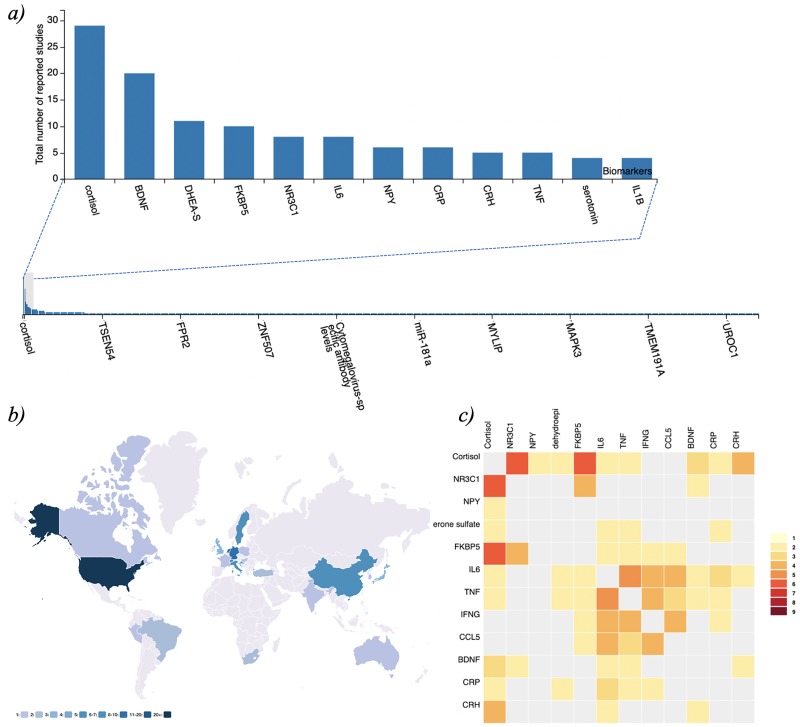
PTSDDM - Biomarker Data and Integrated Metadata: a) Frequency plot of biomarkers captured in the current version of the PTSDDB , b) Geographical map displaying locations of institutions in the curated literature, and c) Heatmap visualization showing the frequency of individual biomarkers studied together in the same articles curated in the PTSDDB. Descriptions of these visualizations are outlined in the Supplementary Information, and these figures can be dynamically explored at https://ptsd.scai.fraunhofer.de/frequencies and https://ptsd.scai.fraunhofer.de/literature.

#### PTSDDB implementation

The application was implemented following a model-view-controller (MVC) software architecture. The back-end is written in Python using the Django web framework technology (https://www.djangoproject.com/). Django embraces the MVC paradigm by storing the data into a MySQL relational database controlled by views that are responsible for querying the database and rendering its content to the users. The front-end renders interactive visualizations using a collection of powerful Javascript libraries: D3.js (https://d3js.org/), C3.js (http://c3js.org/), DataTables (https://datatables.net/) and DataMaps (https://datamaps.github.io/). Because the main goal of the web application is data exploration and visualization, the front-end is powered by Bootstrap, thereby retaining full compatibility with a broad range of devices (e.g. smartphones, tablets, laptops, etc.). Finally, PTSDDB is complemented with a RESTful API documented with an OpenAPI specification (https://www.openapis.org).

## Results and discussion

The PTSDDB is an interactive database that catalogs information on more than 900 physiological markers, extracting data from over 100 manuscripts. The current PTSDDB demonstrates our ability to successfully capture and organize large amounts of knowledge around PTSD physiological markers reported in the literature, using this information to support the creation of interactive data visualizations. By expanding on the PTSDDB in the future, we aim to enable the broad investigation of biomarkers implicated in PTSD pathogenesis.

### Biomarkers overview

**Figure 2:**
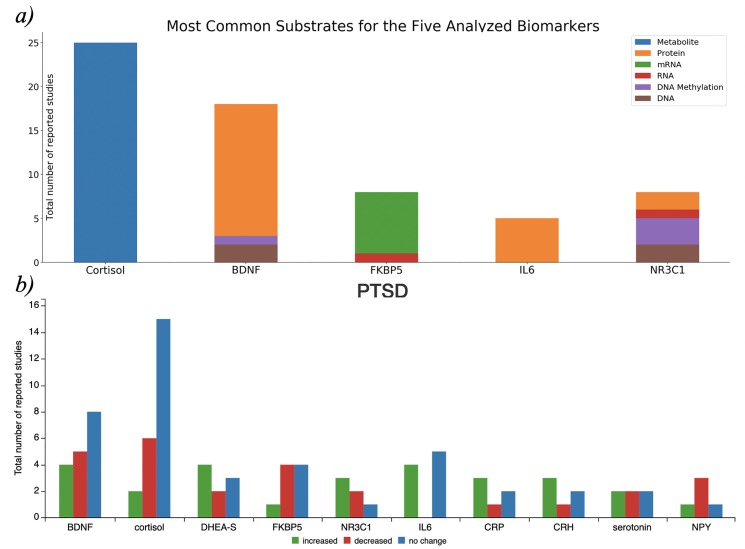
a) Biological substrates of the five most frequently reported biomarkers in PTSDDB when they are studied as a metabolite, protein, or RNA. The source code to reproduce this figure is available at https://github.com/ddomingof/PTSDDB-Resources. b) Relative changes in the ten most common biomarkers captured in the database. This figure can be explored interactively at https://ptsd.scai.fraunhofer.de/relative_changes.

The first page, ‘biomarkers overview’, presents an overview of the biomarker knowledge available in the database, depicted by the frequency of captured biomarkers across studies (Figure 1a), the biofluids in which they were measured, the relative changes reported and the biological substrates captured (e.g. DNA, RNA and protein; Figure 2a).

### Direction of change by biomarker

Also, the second page of the PTSDDB provides information on ‘direction of change by biomarker’ with visualizations that summarize the directionality of biomarker findings (i.e. whether a biomarker was observed to be increased, decreased or unchanged in cases vs controls). For example, Figure 2b summarizes the reported changes in 10 of the most common biomarkers captured in this database. To better contextualize the direction of change, the functionality of this page allows users to assess changes based on the biological substrate, where the biomarker was measured, as well as a dedicated page to explore metadata information, which will subsequently be described.

The current version of the PTSDDB provides a ‘proof-of-concept’ that such visualizations can be successfully created and enable users to interact with articles and data curated in the PTSDDB. The next iteration of the PTSDDB will include a comprehensive landscape analysis of the PTSD biomarker literature so that these visualizations will facilitate researchers’ ability to investigate and critically evaluate the metadata information. For instance, users may glean important information by evaluating and comparing studies around factors that may impact reported findings, such as study type (cross-sectional vs longitudinal), sample size (e.g. number of subjects, controls, etc.), study population (military vs civilian, men vs women) and experimental methods (e.g. how was the biomarker measured).

### Study metadata

While abstracts and results sections summarize the essential findings in biomarker publications, capturing the level of evidence supporting single biomarkers requires information such as sample size or statistical measurements (e.g. mean, standard deviation, etc.), and these data are often unstructured in the form of figures or supplementary files. By storing and cataloging biomarker-related metadata, and then exposing it to the user via the ‘study metadata’ page, the PTSDDB enables users to easily access and query this type of information. In contrast to the previously described information, this page focuses on the sparse world of clinical metadata, which is crucial to compare various study designs that are often complex and heterogeneous in nature. Here, users can first search for studies containing a particular biomarker and then inspect associated metadata (e.g. type of study, duration, challenge, trauma, sample size, assay, diagnostic criteria, etc.) for further analysis. Additionally, users can filter the studies by the specific application of the putative biomarker (i.e. diagnostic, prognostic, risk and stratification), allowing for more precise inquiries. Finally, a quick search box lists the biomarkers analyzed in a given study in order to facilitate the linkage between the meta information displayed in this page with the rest of the pages in PTSDDB (e.g. ‘biomarkers overview’ or ‘direction of change by biomarker’), which are focused on providing a comprehensive overview of the results reported in the studies.

### Literature analysis

Biomarker research is often driven by current trends, technologies and specific hypotheses related to domain expertise. Currently, the increasing quantity of data, information and knowledge makes it incredibly complicated for researchers to stay abreast of all new studies published in a given area of interest. Thus, it is essential to provide researchers with an overview of what biomarkers have already been investigated as well as how the study was conducted. This information not only allows scientists to be aware of what has been studied but also may encourage collaboration among those working on similar hypotheses. To provide an overview of the literature included in the database and foster new research, PTSDDB includes a page with novel visualizations, ‘literature analysis’, illustrating which biomarkers are frequently studied together; where, how, and when were the studies were conducted; or in which biological substrates the biomarkers were measured. First, a table displays the main article information: PubMed-ID, title, journal, authors and year of publication. Second, map-based visualizations represent the geographical distribution of the analyzed articles to help identify PTSD-focused research hubs in the USA and across the globe, which may help identify collaborative opportunities for biomarker replication and validation (Figure 1b). Third, a histogram of the years when the articles included were published (Supplementary Information). Finally, two different heatmaps depict which biomarkers are frequently reported together in publications (Figure 1c) and which are studied in the same bio-fluid (Supplementary Information). By exploring this, we can investigate which combinations of biomarkers are most frequently studied in concert (e.g. cortisol and dehydroepiandrosterone sulfate cortisol and *NR3C1* were measured together in five of the studies that we have included so far in the database) or which biological substrates have been used together in clinical studies.

**Figure 3:**
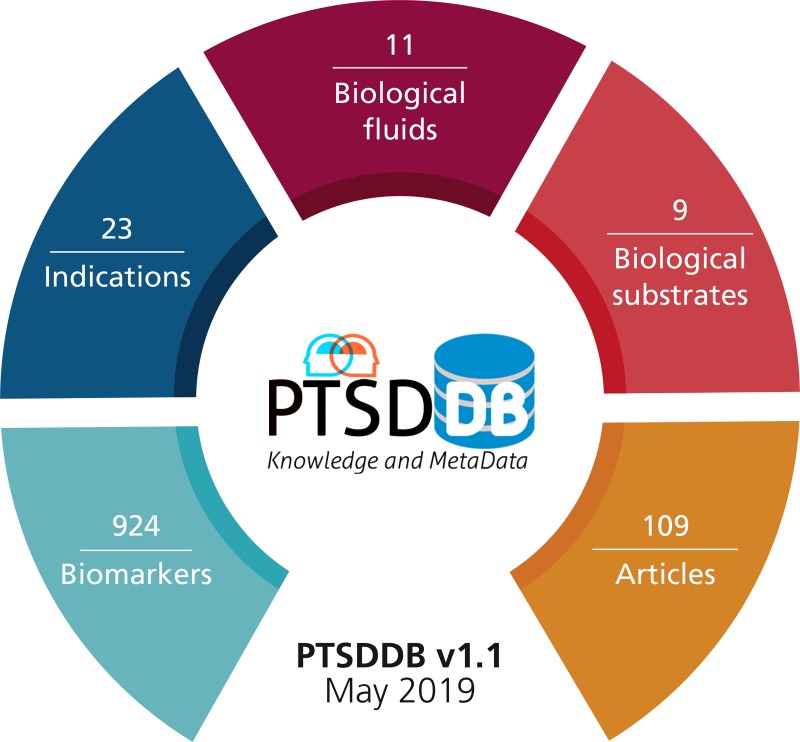
Content of the current version of PTSDDB (May 2019): the database contains 109 articles, 924 biomarkers, 23 indications or distinct manifestations of PTSD, 11 biological fluids, and 9 substrates in which the biomarkers were tested.

### Database content

Since PTSDDB contains a large number of variables and metadata (Figure 3), it is an arduous task to implement interactive visualizations for every possible database query. Therefore, the last page, ‘database content’, contains a RESTful API (https://ptsd.scai.fraunhofer.de/swagger-ui) that exposes the database as well as a summary table of the database, providing both interactive and programmatic interfaces to query, browse and navigate its content. The API is the gateway for researchers who are interested in data models that cannot be accessed through the interactive visualizations presented before (i.e. ancestry information, assay details and calculations, statistical information and inclusion/exclusion criteria). This enables researchers to access specific information extracted from the study, ranging from inclusion/exclusion criteria (e.g. type of medication excluded, comorbidities excluded, etc.) to details about the equipment used in the study (e.g. machine, brand, limits of detection, etc.). Furthermore, users can access associated statistics (e.g. means, standard deviations, *P-*values and fold changes calculated when comparing the biomarker in cases vs controls) in order to conduct or complement future meta-analyses. Finally, the API handles advanced database queries for extracting biomarker information that can be used to conduct complementary bioinformatics analyses as outlined by Zhang *et al*. in the context of colorectal cancer.

## Conclusion

The PTSDDB organizes knowledge in the field of PTSD to provide a review of the literature, bringing together results from different studies so that researchers can evaluate results of single biomarkers, understand how they were measured, in what population and in what clinical contexts of use. This first version of the PTSDDB involved significant curation and harmonization of information from disparate biomarker studies and related literature and storing this information in a database. In the future, we plan to integrate PTSDDB into Brain Commons (https://www.braincommons.org), a big data cloud-based platform for computational discovery designed with user-friendly tools so that the PTSDDB can be regularly updated and openly shared with the research community. In summary, to the best of our knowledge, the PTSDDB is the first resource designed to catalog biomarker knowledge and metadata in PTSD and is complemented with a comprehensive web application that provides interactive visualizations and tools to query the cataloged knowledge. As this resource expands to capture all known knowledge around PTSD biomarkers, we aim to facilitate more formal meta-analyses so that robust conclusions may be drawn around the state of the field, in turn leading to new hypotheses for future studies.

## Supplementary Material

PTSDDB_-_Supplementary_baz081

## Notes

**Database URL:** https://ptsd.scai.fraunhofer.de
